# The Cognitive Profile in Adolescents With Anorexia Nervosa and the Relationship With Autism and ADHD: A Pilot Study

**DOI:** 10.1002/erv.3168

**Published:** 2024-12-28

**Authors:** Sandra Rydberg Dobrescu, Karin Dahlin, Louise Karjalainen, Annelie Bördal Montonen, Helena Klint, Ingrid Stenberg, Gunilla Paulson Karlsson, Elisabet Wentz

**Affiliations:** ^1^ Gillberg Neuropsychiatry Centre Institute of Neuroscience and Physiology University of Gothenburg Gothenburg Sweden; ^2^ The Eating Disorders Centre for Children and Young Adults Sahlgrenska University Hospital Gothenburg Sweden; ^3^ Department of Psychiatry and Neurochemistry Institute of Neuroscience and Physiology University of Gothenburg Gothenburg Sweden; ^4^ Region Västra Götaland Dept. of Neuropsychiatry Sahlgrenska University Hospital Gothenburg Sweden

**Keywords:** anorexia nervosa, childhood, neuropsychology, psychological testing

## Abstract

**Objective:**

We aimed to examine the cognitive profile in adolescents with anorexia nervosa (AN) and its association with traits of autism spectrum disorder (ASD) and ADHD. In addition, resemblance in the cognitive profile between youths with AN and their parents was explored.

**Methods:**

Adolescent females with acute AN (*n* = 20) and a healthy comparison group (*n* = 28) completed neuropsychological tasks of set‐shifting (Trail making test, Wisconsin Card Sorting Test) and central coherence (Rey Complex Figures Task, Group Embedded Figures Test, object assembly subtest). In the AN group, mothers and fathers (*n* = 31) also completed the neuropsychological tasks. Traits of ASD and ADHD were assessed. The AN group was reassessed after weight gain.

**Results:**

Weight‐restored AN adolescents scored higher on the Group Embedded Figures Test than a comparison group (*p* < 0.001). No other set‐shifting and central coherence differences were found across groups. A father‐child correlation emerged in the object assembly subtest (*r* = 0.53, *p* = 0.035). ASD and ADHD traits were common in the AN group and not only related to starvation. No associations were found between neuropsychological deficits and traits of ASD and ADHD.

**Conclusions:**

Scant support was found for weaker central coherence in weight‐recovered adolescents with AN. Set‐shifting impairments could not be observed in young females with acute AN or after weight recovery.

**Summary**

- Weak central coherence was found in weight‐recovered adolescent females with anorexia nervosa (AN); a finding which is in line with previous speculations regarding the endophenotype of AN.
- Some support was found for a father‐daughter association regarding central coherence.
- Set‐shifting deficits could not be detected in the young females with a short duration of AN.

## 1 Introduction

Anorexia nervosa (AN) is a severe psychiatric disorder, predominantly affecting adolescent females. It is characterised by extensive weight loss, restrictive eating patterns and a distorted body image (American Psychiatric et al.). AN usually has its onset during adolescence and is associated with serious medical complications during the course of the disease if not treated (Fayssoil, Melchior, and Hanachi 2021). Over the past 2 decades there has been increasing interest in underlying cognitive markers in individuals with AN that may represent a vulnerability to the disorder. Adult AN populations have shown inefficient cognitive processing including weak central coherence (Lang et al. 2014a) and impaired set‐shifting (Roberts et al. 2007). Weak central coherence refers to the tendency to prioritise focussing on details and difficulty processing information in a global context. Weak central coherence might contribute to poor treatment outcomes as it has been linked to exaggerated obsessive‐compulsive and perfectionistic personality traits hindering behavioural change (Lang et al. 2014a). Set‐shifting refers to the ability to shift back and forth between multiple tasks, operations or mental sets (Miyake et al. 2000). Set‐shifting difficulties have been suggested to underlie the rigid and inflexible thinking seen in patients with AN. An inflexible thinking style may interfere with a patient's ability to accept behavioural and cognitive change during therapy (Treasure et al. 2013). In adults, impaired set‐shifting and central coherence persist after weight restoration and are also found in unaffected adult family members, suggesting they may be cognitive endophenotypes in AN (Holliday et al. 2005; Lang, Treasure, and Tchanturia 2016a; Tenconi et al. 2010). Regarding younger populations with AN, the available data on central coherence and set‐shifting are inconclusive; some studies report impaired set‐shifting (Lang et al. 2015; Wu et al. 2014) and central coherence (Lang et al. 2014a) in adolescents with AN, while others reveal no deviances compared with healthy controls (Bentz et al. 2017; Leslie et al. 2021; van Noort et al. 2016; Shott et al. 2012). As the inefficiencies appear to be less pronounced in adolescents with AN, it has been suggested that the impairments are a consequence of starvation and/or longer duration of AN (Lang et al. 2014b; Saure et al. 2020).

An overlap has been demonstrated between AN and attention deficit hyperactivity disorder (ADHD) and autism spectrum disorder (ASD), and all three conditions seem to share cognitive deficits (Happé et al. 2008; Nickel et al. 2019). Already in the 1980s, AN was suggested to be of neurodevelopmental origin (Gillberg 1983). Since then, there has been growing interest in the relationship between AN and ASD. Patients with AN are often described as socially reserved, displaying rigid and repetitive thoughts and behaviours—characteristics that are also distinctive of ASD (Zucker et al. 2007). More recently, a number of studies have reported elevated ASD traits among individuals with AN (Westwood et al. 2016; Huke et al. 2013), and a subgroup of individuals with AN has been found to fulfil the diagnostic criteria of ASD (Anckarsäter et al. 2012). A recent systematic review on the topic reported autistic traits being overrepresented in AN and apparently remaining stable over time (not only related to low body weight) (Boltri et al. 2021). Elevated ASD traits in AN have been linked to poorer outcomes considering overall functioning, longer duration of AN and longer treatment periods (Saure et al. 2020; Nielsen et al. 2015).

In addition, individuals with higher ASD traits report poorer experiences of treatment and may be at increased risk of admittance to inpatient care (Nimbley et al. 2024; Zhang et al. 2022). The importance of research in the field of ASD and EDs, including screening procedures and adaptations into clinical practice, has recently been highlighted (Tchanturia 2022). Furthermore, the relationship between the cognitive profiles of individuals with EDs and ASD was addressed as one of several key components in need of further investigation (Tchanturia 2022).

The relationship between ADHD and AN is less studied. The association between Bulimia nervosa (BN) and Binge‐eating disorders (BED) on the one hand and ADHD on the other has been repeatedly demonstrated (Nazar et al. 2016; Seitz et al. 2013). Especially the binging/purging subtype of AN has been associated with ADHD (Wentz et al. 2005; Svedlund et al. 2017). Svedlund et al. (Svedlund et al. 2017) found that the frequency of ADHD symptoms was as high in adults with AN (binging/purging subtype) as in adults with BN. The relationship between binging/purging behaviour and ADHD is driven at least in part by impulsivity, a central feature of both conditions (Seitz et al. 2013).

To summarise, it remains unclear whether poor set‐shifting and weak central coherence are present in young individuals with AN of short duration. Furthermore, in young populations, a putative association between cognitive deficits and co‐occurring AN, ASD, and ADHD needs to be further explored to improve the knowledge of a possible link in this age group.

### 1.1 Aims

The aims of the present study were to investigate adolescent females with AN.

1. regarding central coherence and set‐shifting in the acute phase and after weight restoration, and to contrast them with a healthy comparison group;
2. regarding the resemblance between their neuropsychological profile and that of their parents;
3. with respect to associations between possible cognitive impairments and traits of ASD and ADHD.

We hypothesised that the AN group, when contrasted with a comparison group, would exhibit poorer results on tasks measuring set‐shifting and central coherence and that these impairments would persist after weight restoration. Secondly, we expected the cognitive profiles of the adolescents to be associated with the cognitive profiles of their parents. Thirdly, we hypothesised that ASD traits and ADHD symptoms would be associated with underlying cognitive deficits and be more prevalent in the AN group compared with a comparison group.

## 2 Methods

### 2.1 Participants

#### 2.1.1 Anorexia Nervosa Group

Twenty adolescent females with AN seeking treatment at a specialised unit for outpatient treatment (the Eating Disorder Centre for Children and Young Adults, Sahlgrenska University Hospital, Gothenburg, Sweden) were recruited during the acute phase of the illness while underweight (AN‐A group). One or both biological parent(s) were also recruited. All of the individuals with AN were diagnosed according to the DSM‐IV criteria by a psychiatrist (American Psychiatric et al. 2000). The inclusion criteria were defined as (1) a current DSM‐IV diagnosis of AN, (2) age between 12 and 18 years, and (3) Swedish‐speaking. A known intellectual disability (IQ below 70) was the only exclusion criterion. After recruitment, the young females completed a battery of neuropsychological tests and self‐reports. A follow‐up assessment was conducted approximately 1 year later when the participants were no longer classified as underweight (< age18: Body mass index (BMI) standard deviation score > −2; ≥ age 18: BMI > 18.5 kg/m^2^ (American Psychiatric et al.)). The weight‐recovered group is referred to as the *‘AN‐WR group’.* The majority of the AN‐WR group was still in treatment. One participant declined participation in the follow‐up assessment leaving 19 individuals to constitute the AN‐WR group.

#### 2.1.2 Comparison Group

A healthy comparison group (COMP) consisting of 29 female adolescents was recruited from three different schools in Gothenburg. The headmasters were contacted and informed about the study and its procedure. Information about the study was then sent by e‐mail to the students' parents. Written information about the study and informed consent forms were sent to the students' parents. The inclusion criteria were (1) absence of current or previous eating disorder (ED), and (2) age between 12 and 18 years. The exclusion criterion was known intellectual disability. One recruited student reported a history of AN and was therefore excluded from the analysis, leaving 28 female adolescents in the COMP group.

#### 2.1.3 AN Parent Group

One or both biological parent(s) of the AN adolescents were recruited to the study. Parents of 18 of the individuals in the AN group (mothers *n* = 15, fathers *n* = 16) took part in the study.

### 2.2 Procedures

**FIGURE 1:**
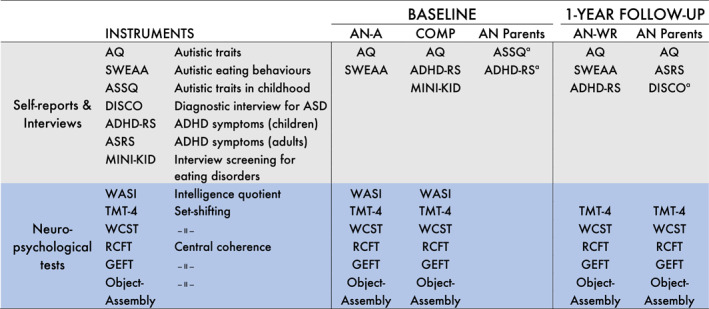
Instruments administered at baseline and at the 1‐year follow‐up in the anorexia nervosa (AN) group, the comparison group and the AN parent group. Each instrument is presented with a short description of what it measures. Parental reports of ADHD symptoms were used at baseline in the AN group to assess current and childhood symptoms. ADHD‐RS, ADHD rating scale IV; AN‐A, Acute anorexia nervosa; AN‐WR, Anorexia nervosa weight restored; ASD, Autism spectrum disorders; ASRS, ADHD Self Report Scale; ASSQ, The Autism Spectrum Screening Questionnaire; AQ, the autism spectrum questionnaire; COMP, Comparison cases; DISCO, The Diagnostic Interview for Social and Communication disorders; GEFT, Group Embedded Figures Test; MINI‐KID, Mini International Neuropsychiatric Interview for Children and Adolescents; RCFT, Rey Complex figure test; SWEAA, the Swedish eating assessment for autism spectrum disorders; TMT‐4, Trail making Test 4; WASI, Wechsler Abbreviated Scale of Intelligence; WCST, Wisconsin Card Sorting Test. ^a^Parental report covering the child's symptoms.

At baseline, the AN‐A group was assessed with a neuropsychological test battery to measure general intelligence, set‐shifting and central coherence (instruments described below). The participants also completed self‐report questionnaires (Figure 1). The whole procedure, including questionnaires, required approximately 2–2.5 h. When the test battery was too tyring for the individuals with acute AN to complete in one session, the tests were administered on two occasions, 1 week apart. The weight and height of the participants were measured on the day of testing. Body mass index (BMI) was calculated as kilogrammes per metres squared. BMI *Z* scores were calculated to adjust for the child's age and sex (Must et al. 2006). Age‐based height‐weight tables based on a Swedish population were used as a standard reference for BMI (He, Albertsson‐Wikland, and Karlberg 2000). Information on AN duration (in months) was collected at the first visit to the unit for outpatient treatment at the Eating Disorder Centre for Children and Young Adults. Psychiatric morbidity was collected from the medical records of the AN participants. Participants who were no longer patients at the Eating Disorder Centre for Children and Young Adults at the time of the follow‐up self‐reported their psychiatric morbidity. The AN parent group completed questionnaires and interviews covering the child's symptoms (Figure 1).

The COMP group also completed the battery of neuropsychological tests and self‐reports. They were assessed at school by a clinical psychologist. The students were screened for current or previous eating disorders (EDs). Weight and height were self‐reported by the students at the time of the assessment. In addition, the researchers were in contact with the school health nurses regarding whether the student exhibited any deviances in weight causing suspicion of AN.

The AN‐WR group was reassessed 1 year after the baseline assessments. The adolescents were also screened for ADHD. Participants who were not weight‐restored after 1 year were contacted again after approximately one and a half years for the follow‐up. The mean duration from baseline to follow‐up was 495 days (SD: 167, range: 347–858 days). Around the time of the follow‐up, the parents of the individuals with AN were subjected to neuropsychological assessments (WASI excluded) and completed the adult self‐reports. The neuropsychological tests were administered by a clinical psychologist. Five psychologists from the Eating Disorder Centre for Children and Young Adults were involved in the test administration. At the follow‐up, all parents were asked to take part in a structured in‐depth interview regarding autistic traits during childhood. Not all parents agreed to participate, mainly because the interview was time‐consuming. For ethical reasons we only asked the parent(s) twice to participate in the interview. Ten parents (mothers *n* = 5, fathers *n* = 5) of 10 participants were interviewed. Baseline data were collected from August, 2013, to March, 2016. The follow‐up assessments (including those of parents) were completed in December, 2018.

### 2.3 Instruments

#### 2.3.1 Neuropsychological Assessments

##### 2.3.1.1 Intellectual Ability

All participants completed the four core subtests (Block Design, Matrix reasoning, Vocabulary and Similarities) that constitute the Wechsler Abbreviated Scale of Intelligence, WASI (D 2011). The WASI provides intelligence estimates in perceptual and verbal reasoning as well as a full‐scale IQ.

##### 2.3.1.2 Set‐Shifting Tasks

Cognitive set‐shifting was measured using the Delis and Kaplan Executive Function System (Delis et al. 2001) (DKEFS), the Trail Making Test Condition 4 (TMT‐4) and the Wisconsin Card Sorting Test (WCST). The TMT‐4 was used as a primary outcome measure in the present study. The TMT‐4 was administered using pen and paper and prompts individuals to connect a sequence of numbers and letters in ascending order as quickly as possible (e.g., 1‐A‐2‐B). The time required to complete the task is the indicator of set‐shifting ability. The computerised version of the ‘Wisconsin Card Sorting Test’ (WCST) (Heaton et al. 1993), in which the proband has to match cards according to changing rules, was used. Participants are given feedback after each trial; however, the classification rule (colour, shape, number) changes every 10 cards and the proband must quickly adapt to the new rule through implicit learning.

##### 2.3.1.3 Central Coherence Tasks

Central coherence was measured with the Rey Complex Figure Test (RCFT) (Osterrieth 1944) and the Group Embedded Figures Test (GEFT) (Oltman, Raskin, and Witkin 1971). A central coherence index (CCI) was obtained from calculating the order of construction index and the style index according to Booth (2006) (Booth 2006) and Lopez (2008) (Lopez et al. 2008). The order of construction assesses the order in which the elements are drawn (detailed, global) and the style of drawing (fragmented, coherent). The order of construction index, the OCI (range: 0–3.3), together with the style index, the SI (range: 0–2), constitute the CCI. The CCI ranges from 0 to 2 and provides information on a detailed or global approach to the task. A higher CCI score indicates a better global or holistic processing ability. We used the slightly modified scoring instructions described by Lang et al. to enable comparison of our data with normative CCI data in eating disorders (Lang et al. 2016b). The GEFT requires the participant to identify simple figures that are hidden within a progressively more complicated figure. The GEFT was administered using pen and paper. The test assesses the ability to disembed certain information from surrounding irrelevant information. The number of correctly traced figures, range 0–18, works as an outcome measure and higher scores indicate a more detailed approach (Witkin et al. 2002). An additional measure of central coherence was ‘the object assembly’ subtest from the Wechsler adult/child intelligence scale III. Participants are asked to complete five jigsaw puzzles depicting familiar objects. It requires the ability to disregard concrete details in the construction of a coherent ‘whole’. The main outcome measure is the time taken to complete each puzzle. Scaled scores were used where a higher score indicates shorter time and better global integration. A second researcher co‐rated 10% of the CCI data for each group. The inter‐rater agreement (Cohen's kappa) was 0.87, indicating excellent agreement.

##### 2.3.1.4 ED Screening in the COMP Group

The ED module of the Mini International Neuropsychiatric Interview for Children and Adolescents (MINI‐KID) was used to screen the students in the COMP group for EDs. MINI‐KID is a structured clinical diagnostic interview designed to assess the presence of current *DSM‐IV* and *ICD‐10* psychiatric disorders in children and adolescents aged 6–17 years (Sheehan et al. 2010).

##### 2.3.1.5 Self‐Report Questionnaires: AN and COMP Group

*The autism spectrum questionnaire (AQ)* is a self‐report questionnaire based on the autism spectrum symptomatology and was first introduced by Baron‐Cohen and colleagues (Baron‐Cohen et al. 2001). It contains 50 items based on five different areas with a suggested cutoff of 32 points to identify clinically significant levels of autistic traits (Baron‐Cohen et al. 2001). *The Swedish Eating Assessment for Autism spectrum disorders* (SWEAA) is a self‐report questionnaire targeting typical eating and mealtime problems in individuals with ASD and normal intelligence (Karlsson, Råstam, and Wentz 2013). The SWEAA is based on 60 items divided into a number of subscales, all targeting different areas of deviant eating behaviours: A. Perception; B. Motor control; C. Purchase of food; D. Eating behaviour; E. Mealtime surroundings; F. Social situation at mealtime; G. Other behaviours associated with disturbed eating; H. Hunger/satiety; I. Simultaneous capacity, and J. Pica. The SWEAA has shown high levels of reliability in terms of Cronbach's alpha and good test‐retest ability (Karlsson, Råstam, and Wentz 2013). The total score was calculated and a cutoff value for overall eating behaviour of 12 points was used. The two subscales ‘F. Social situation at mealtime’ and ‘I. Simultaneous capacity’, discriminate best between individuals with ASD and healthy controls (best two subscale discriminating score, BTSD score) and a mean score of the two subscales was used. The BTSD score has a suggested cutoff of 10 points.

##### 2.3.1.6 Questionnaires and Interviews Completed by the AN Parent Group

*The Autism Spectrum screening questionnaire (ASSQ)* is a screening questionnaire for autism (Ehlers, Gillberg, and Wing 1999). It comprises 27 items and is rated on a three‐point scale, (0 = indicating normality, 1 = some abnormality, and 2 = definite abnormality), with a possible maximum score of 54 points. Eleven items cover topics regarding social interaction, six target communication problems and five refer to restricted and repetitive behaviour. The remaining five items embrace motor clumsiness and other associated symptoms including motor and vocal tics. Cutoff scores of 19 or more for the parental version have been recommended in Sweden for screening to identify ASDs in children (Ehlers, Gillberg, and Wing 1999). To better capture the phenotype of autism in females, 18 items were added by Kopp and colleagues and constitute the ASSQ‐REV (Kopp et al. 2011). *The ADHD Rating Scale IV (ADHD‐RS)*, a semi‐structured interview measuring ADHD symptoms (DuPaul, Anastopoulos, and Reid 1998), was administered to the parents at baseline regarding their child's symptoms. *The Diagnostic Interview for Social and COmmunication disorders (DISCO)* (Wing et al. 2002) is a standardized interview used to assist clinicians in the diagnostic work‐up and care of persons with ASD. The interview takes 2–4 h and is intended for interviewing a person who has known the individual with suspected ASD from early childhood.

##### 2.3.1.7 Self‐Report Questionnaires: AN Parent Group

The AN parents also reported on their own traits by completing the AQ and *the ADHD Self‐Report Scale* (ASRS‐ v1.1). The ASRS is one of various self‐report measures developed to assess current manifestations of ADHD symptoms in individuals aged 18 years or older (Kessler et al. 2005). It covers a checklist of inattentive, hyperactive, and impulsive symptoms including 18 items to measure the symptoms specified in the DSM‐IV. Respondents are required to use a five‐item Likert scale to indicate the frequency of the occurrence of symptoms (0 = never; 1 = rarely; 2 = sometimes; 3 = often; 4 = very often). The first six questions (Part A) were found to be the most predictive of symptoms consistent with ADHD. If four or more questions of Part A were answered in the affirmative, this signals symptoms highly consistent with ADHD in adults (Adler et al. 2006). A global score (range 0–72) was also calculated.

### 2.4 Statistical Analysis

Analyses were performed using SAS v9.3 (Cary, NC). Mainly non‐parametric tests were used, due to the data not being normally distributed. Differences in baseline characteristics between the AN and the COMP group were examined with Fisher's non‐parametric permutation test for continuous variables and Fisher's exact test for dichotomous variables. For comparison between the groups regarding primary and secondary outcome measures, the Mantel‐Haenszel Chi Square Exact test was used for ordered categorical variables and Fisher's non‐Parametric Permutation test for continuous variables.

For analysis of change within the AN group, Fisher's non‐parametric permutation test for paired observations was used. Spearman's correlation coefficient was used for all correlation analyses. Primary and secondary outcome measures were defined before the start of the study and were therefore not adjusted for multiple testing. Missing data regarding the *G* scale of the SWEAA applied to three individuals at baseline and two at follow‐up. The missing values in the ‘G scale’ of the SWEAA questionnaire were replaced using stochastic imputation.

All tests were two‐tailed and conducted at a significance level of 0.05.

The following baseline variables were analysed as predictors of weight recovery 1 year after baseline assessments: age, Z‐BMI, duration of AN in months, IQ (WASI score), neuropsychological test scores of the participants, neuropsychological test scores of the parents, traits of ASD and ADHD. The effect of baseline variables on weight recovery were analysed using univariable logistic regression with Firth correction to mitigate cases of complete separation. Variables with *p*‐values < 0.10 in the univariable analysis were then included in a multivariable logistic regression model (also with Firth correction). The model was evaluated using Tjur's discrimination index. Confidence intervals were calculated using non‐parametric bootstrap with *M* = 200 bootstrap replicates. To assess the variable importance for explaining weight recovery versus non‐recovery 1 year after baseline, the Tjur discrimination index was decomposed amongst the model variables using Shapley values, such that the sum of contributions from each individual variable adds up to the total Tjur discrimination index of the full model. Additionally, the area under the receiver‐operator‐curve (AUC) was computed using leave‐one‐out cross validation for the significant (at 0.10‐level).

### 2.5 Ethical Approval

The study was approved by the Regional Ethical Review Board at the University of Gothenburg (reg. no. 395‐12). Detailed information about the study and the procedure was given to all participants and their parents before participation. All participants and/or their parents provided written informed consent. In Sweden, individuals may participate in research without parental consent from the age of 15.

## 3 Results

Participant demographics and clinical characteristics are presented in Table 1. There was no significant difference in age and estimated level of intelligence quotient between the AN‐A group and the COMP group. At baseline, the AN‐A group presented a significantly lower BMI *Z* score than the COMP group (*p* < 0.0001). The BMI *Z* score had increased significantly in the AN group from baseline to the 1‐year follow‐up (*p* = < 0.001) but remained significantly lower compared with the COMP group (*p* = 0.005).

**TABLE 1:** Demographics and clinical characteristics at baseline and at the 1‐year follow‐up.

	Baseline				1‐year follow‐up		
	AN‐A	COMP	AN‐A versus COMP	Effect size	AN‐WR	AN‐WR versus COMP	Effect size
	*n* = 20	*n* = 28	*p* value		*n* = 19	*p* value	
Age, mean (SD)	14.2 (1.3)	14.8 (1.5)	0.17	0.389	15.5 (1.4)	0.13	0.476
BMI (kg/m^2^), mean (SD)	16.5 (1.0)	20.2 (2.0)	< 0.0001	2.17	19.0 (1.2)	0.022	0.692
BMI *Z* score, mean (SD)	−1.29 (0.55)	−0.017 (0.61)	< 0.0001	2.13	−0.53 (0.5)	0.005	0.882
Duration of AN in months, mean (SD)	14.7^a^ (9.4)	n.a.	n.a.	n.a.	n.a.	n.a.	n.a.
Any psychiatric disorder excluding eating disorders, *n* (%)	4^b^ (21%)	n.a.	n.a.	n.a.	10^c^ (53%)	n.a.	n.a.
IQ from WASI, mean (SD)	105.2 (9.0)	102.1 (6.2)	0.19	0.396	n.a.	n.a.	n.a.

*Note:* Statistics are based on Fisher's Non‐Parametric Permutation Test.

Abbreviations: AN‐A, Acute anorexia nervosa; BMI, Body mass index; COMP, Comparison; IQ, Intelligence quotient; n.a., not applicable; SD, Standard deviation; WASI, Wechsler Abbreviated Scale of Intelligence.

^a^ Data missing for six participants.

^b^ Affective disorder (*n* = 3), Obsessive‐compulsive disorder (*n* = 1).

^c^ Affective disorder (*n* = 7), Anxiety disorder (*n* = 2), ADHD (*n* = 2).

### 3.1 Neuropsychological Assessments

There were no significant differences across the two groups at baseline regarding any of the neuropsychological test results (Table 2). At the 1‐year follow‐up, performance on the RCFT accuracy scores and the GEFT scores was significantly higher in the AN‐WR group compared with the COMP group (*p* = 0.012 and *p* = 0.0008, respectively; note: only baseline data were available for the COMP group). The GEFT scores increased in the AN group between the baseline and the follow‐up assessment, indicating enhanced detail processing. Regarding the WCST scores, perseverative errors and responses declined significantly between the two assessments in the AN group (Table 3).

**TABLE 2:** Neuropsychological test results of adolescents with acute anorexia nervosa and after weight restoration contrasted with a healthy comparison group.

	Baseline					1‐year follow‐up			
	AN‐A (*n* = 20)	COMP (*n* = 28)	Difference between groups	AN‐A versus COMP	Effect size	AN‐WR (*n* = 19)	Difference between groups	AN‐WR versus COMP	Effect size
	Mean (SD)	Mean (SD)	Mean (95% CI)	*p* value		Mean (SD)	Mean (95% CI)	*p* value	
TMT‐4	10.0 (1.62)	10.4 (2.2)	−0.429 (−1.583; 0.727)	0.5	0.213	9.6 (2.3)	−0.850 (−2.20; 0.545)	0.24	0.372
WCST total score (raw score)	70.0 (12.4)	67.7 (8.5)	2.32 (−3.73; 8.50)	0.48	0.223	70.2 (8.4)	2.50 (−2.87; 7.67)	0.35	0.289
WCST perseverative responses (raw score)	18.4 (24.9)	13.7 (13.8)	4.65 (−6.67; 14.78)	0.49	0.240	9.41 (5.16)	−4.30 (−11.67; 1.60)	0.25	0.371
WCST perseverative errors (raw score)	15.6 (18.4)	12.4 (11.3)	3.19 (−5.58; 11.00)	0.52	0.216	8.59 (4.93)	−3.80 (−9.90; 1.20)	0.21	0.396
WCST number of categories (raw score)	5.21 (1.81)	5.64 (1.10)	−0.432 (−1.250; 0.455)	0.38	0.298	5.5 (1.37)	−0.113 (−0.800; 0.667)	0.83	0.092
GEFT (raw score)	10.6 (4.8)	8.8 (4.6)	1.76 (−1.07; 4.50)	0.22	0.371	13.3 (3.5)	4.53 (2.08; 7.09)	<0.001	1.06
RCFT copy condition									
Accuracy (raw score)	30.9 (3.1)	30.0 (4.1)	0.832 (−1.292; 3.000)	0.47	0.221	32.4 (1.41)	2.38 (0.50; 4.36)	0.012	0.711
Order	1.74 (0.65)	1.90 (0.57)	−0.164 (−0.516; 0.197)	0.36	0.267	2.04 (0.66)	0.140 (−0.226; 0.503)	0.44	0.226
Style	1.39 (0.39)	1.54 (0.29)	−0.144 (−0.347; 0.056)	0.16	0.420	1.53 (0.50)	−0.003 (−0.231; 0.234)	0.97	0.006
Central coherence index	1.24 (0.37)	1.34 (0.27)	−0.103 (−0.287; 0.087)	0.28	0.320	1.42 (0.36)	0.078 (−0.106; 0.264)	0.40	0.251
Object assembly	10.4 (2.4)	11.4 (2.4)	−1.04 (−2.44; 0.33)	0.15	0.434	11.3 (3.2)	−0.077 (−1.700; 1.583)	0.97	0.028

Abbreviations: AN‐A, Acute anorexia nervosa; AN‐WR, Anorexia nervosa weight restored; CI, Confidence interval; COMP, Comparison; GEFT, Group embedded figures test; RCFT, Rey Complex figures test; SD, Standard deviation; TMT‐4, Trail making test condition 4; WCST, Wisconsin card sorting test.

**TABLE 3:** Change within the anorexia nervosa group on neuropsychological measures from baseline to follow‐up.

	AN‐A (*n* = 20)	AN‐WR (*n* = 19)	Change from AN‐A to AN‐WR		
			Mean (95% CI)	*p* value	Effect size
TMT‐4	10.0 (1.62)	9.6 (2.32)	−0.32 (−1.35; 0.71)	0.59	−0.2
WCST total score (raw score)	70.0 (12.4)	70.2 (8.4)	−2.69 (−5.26; −0.12)	0.047	0.32
WCST perseverative responses (raw score)	18.4 (24.9)	9.41 (5.16)	−3.81 (−7.02; −0.60)	0.031	−0.74
WCST perseverative errors (raw score)	15.6 (18.4)	8.59 (4.93)	−3.38 (6.34; −0.41)	0.037	−0.72
WCST number of categories (raw score)	5.21 (1.8)	5.53 (1.37)	0.063 (−0.536; 0.661)	1.0	0.04
GEFT (raw score)	10.6 (4.8)	13.3 (3.5)	2.47 (0.92; 4.03)	0.0022	0.52
RCFT copy condition					
Accuracy (raw score)	30.9 (3.1)	32.4 (1.41)	1.29 (0.01; 2.57)	0.053	0.44
Order	1.74 (0.65)	2.04 (0.66)	0.31 (−0.151; 0.768)	0.17	0.46
Style	1.39 (0.39)	1.53 (0.50)	0.15 (−0.177; 0.472)	0.35	0.37
Central coherence index	1.24 (0.37)	1.42 (0.36)	0.19 (−0.079; 0.464)	0.15	0.5
Object assembly	10.4 (2.4)	11.3 (3.2)	1.0 (−0.24; 2.24)	0.13	0.41

Abbreviations: AN‐A, Acute anorexia nervosa; AN‐WR, Anorexia nervosa weight restored; GEFT, Group embedded figures test; RCFT, Rey Complex figures test; TMT‐4, Trail making test condition 4; WCST, Wisconsin card sorting test.

### 3.2 Autistic Traits and ADHD Symptoms in the AN Group

The AN group scored significantly higher on the AQ than the COMP group in both the first (*p* = 0.0017) and the second assessment (*p* = 0.02). Furthermore, the mean AQ score did not decrease significantly from baseline to the follow‐up assessment (*p* = 0.21) in the AN group after weight gain (Table 4). In the AN group, 10% (*n* = 2) and 5.3% (*n* = 1) scored above the cutoff for ASD in the first and second assessment, respectively, compared with none in the COMP group.

**TABLE 4:** Autistic traits and ADHD symptoms in the anorexia nervosa (AN) group, the comparison group and the AN parent group.

	AN‐A (*n* = 20)	COMP (*n* = 28)	AN mother (*n* = 15)	AN father (*n* = 16)	AN‐A versus COMP	Effect size	AN‐WR (*n* = 19)	Change from AN‐A to AN‐WR	AN‐A versus AN‐WR	Effect size
	Mean (SD)	Mean (SD)	Mean (SD)	Mean (SD)	p value		Mean (SD)		p value	
**Self‐reports**										
AQ	19.6^a^ (8.6)	12.9 (4.9)^b^	9.87^b^ (4.5)	13.0^b^ (6.2)	0.0017	0.977	17.9^c^ (9.0)	−1.95 (6.35)	0.21	−0.22
SWEAA total score	35.2^d^ (12.1)	n.a.	n.a.	n.a.			26.8 (10.9)	−8.48 (9.75)	0.0022	−0.68
SWEAA BTSD score	32.1^d^ (13.2)	n.a.	n.a.	n.a.			26.6 (14.8)	−5.88 (13.42)	0.092	−0.44
ASRS part A	n.a.	n.a.	1.20^e^ (1.32)	1.31^f^ (1.3)			n.a.			
ASRS total	n.a.	n.a.	24.93 (7.7)	21.88 (7.5)			n.a.			
ADHD‐RS	n.a.	1.80^g^ (1.8)	n.a.	n.a.			4.39 (4.2)			
										
**Parental reports**										
ASSQ	4.4^h^ (3.3)	n.a.								
ADHD‐RS	1.65^i^ (1.73)	n.a.								

Abbreviations: ADHD‐RS, The ADHD rating scale IV; AN‐A, Acute anorexia nervosa; AN‐WR, Anorexia nervosa weight‐restored; ASRS, the ADHD Self Report Scale (cutoff part A: 4); ASSQ, The Autism Spectrum screening questionnaire (cutoff: 19); AQ, the autism spectrum questionnaire (cutoff: 32); COMP, Comparison; n.a., not applicable; SD, Standard deviation; SWEAA, the Swedish eating assessment for autism spectrum disorders (total score cutoff: 12; BTSD score cutoff: 10).

^a^ Two scored above the AQ cutoff.

^b^ None scored above the AQ cutoff.

^c^ One scored above cutoff.

^d^ Data missing for two participants.

^e^ One mother scored above the ASRS cutoff.

^f^ One father scored above the ASRS cutoff.

^g^ Based on 10 individuals from the COMP group.

^h^ Data missing for one participant.

^i^ Data missing for three participants.

Parental reports of autistic traits in the AN‐A group, measured with the ASSQ, were all below the cutoff for ASD (Table 4), and none of the participants fulfiled the criteria for ASD according to the DISCO interviews.

The ADHD‐RS scores at baseline in the AN group were below the cutoff for all participants. At the follow‐up, 10.5% (*n* = 2) of the individuals in the AN‐WR group scored above the cutoff on the ADHD‐RS (both ADHD combined type). Another two adolescents in the AN group had been assigned a diagnosis of ADHD around the time of the follow‐up, and one of them was using stimulants at the follow‐up.

### 3.3 Eating Behaviours Frequently Seen in Individuals With ASD in the AN Group

Eating behaviours frequently seen in individuals with ASD, measured with the SWEAA, in the AN group during the starvation phase and at follow‐up are shown in Table 4. The overall abnormal eating behaviours had decreased significantly from baseline to the 1‐year follow‐up (*p* = 0.0022), as measured by the total SWEAA score. There was no significant decrease in deviant eating behaviours as measured by the SWEAA BTSD‐score (*p* = 0.092) at follow‐up.

### 3.4 Resemblance in Neuropsychological Profile of the Adolescents With AN and Their Parents

There was a significant positive correlation between the object assembly scores of the AN group and the object assembly scores of their fathers (*r* = 0.53; *p* = 0.035). There were no other statistically significant parent‐child correlations (Table S1).

### 3.5 Correlations Between Neuropsychological Measures and Traits of ASD and ADHD

In the AN group, there were no statistically significant correlations between the AQ scores at baseline and the CCI score (*r* = −0.26, *p* = 0.269), the GEFT scores (*r* = −0.28, *p* = 0.224), the Object assembly scores (*r* = −0.36, *p* = 0.118), the TMT‐4 scores (*r* = −0.05, *p* = 0.804), or WCST perseverative errors (*r* = 0.066, *p* = 0.784). At follow‐up, there was a negative correlation between the AQ scores and the WCST perseverative errors (*r* = −0.577, *p* = 0.0153); that is, the higher the AQ scores, the lower the number of perseverative errors. No other significant correlations were found at the follow‐up between central coherence tasks, set‐shifting tasks and AQ scores (CCI: *r* = −0.136, *p* = 0.576; GEFT: *r* = 0.109, *p* = 0.655; Object Assembly subtest: *r* = −0.034, *p* = 0.887; TMT‐4: *r* = 0.220, *p* = 0.365). The set‐shifting data did not correlate with the ADHD‐RS scores at baseline or at the follow‐up (Baseline: TMT‐4: *r* = −0.04, *p* = 0.890; WCST perseverative errors scores: *r* = 0.34, *p* = 0.238; Follow‐up: TMT‐4: *r* = −0.04, *p* = 0.89; WCST perseverative errors: *r* = 0.34, *p* = 0.238).

### 3.6 Correlations Between Duration of AN and Other Variables

There was a significant negative correlation between the object assembly score and the duration of AN (*r* = −0.54, *p* = 0.046). No statistically significant correlations were found between TMT‐4 (*r* = 0.18, *p* = 0.546), WCST perseverative errors (*r* = −0.15, *p* = 0.601), CCI (*r* = −0,12, *p* = 0.677), AQ (*r* = −0.039, *p* = 0.895) or ADHD‐RS (*r* = 0.11, *p* = 0.724) and the duration of AN.

### 3.7 Predictors of Weight Recovery in the AN Group One Year After Baseline Assessments

Predictors of weight recovery 1 year after baseline with *p*‐values < 0.10 in the univariable analysis were: age, Z‐BMI and IQ (WASI score). A higher age, a lower IQ score and a lower Z‐BMI at baseline were predictors of weight recovery 1 year after baseline assessments (Table S2). The multivariable regression revealed that IQ had the highest variable importance (Tjur's discrimination index: 0.32, 95% CI: 0.05, 0.64) as presented in Table S3.

## 4 Discussion

The aim of this study was to examine the cognitive profile in adolescent females with AN, in the acute phase and after weight recovery, and explore its association with traits of ASD and ADHD. An additional aim was to explore a putative resemblance of the cognitive profiles between adolescents with AN and their biological parents. Overall, we found some support for superior detail processing in the weight‐recovered AN group compared with controls. No other statistically significant differences regarding set‐shifting and central coherence were found across groups. The resemblance in cognitive profile between the AN adolescents and their parents was limited to one central coherence outcome, the object assembly task. Moreover, ASD traits and ADHD symptoms were more common in the weight‐recovered AN group compared with healthy adolescents. No associations were found between central coherence or set‐shifting data and AQ scores or between set‐shifting data and ADHD‐RS results.

We hypothesised that young females with AN would present poorer results on tests measuring set‐shifting and central coherence and that these deviances would persist after weight recovery. To some extent this could be confirmed. The weight‐restored AN group scored significantly higher on the GEFT after weight restoration, compared with the COMP group, indicating superior detail processing (one aspect of weak central coherence). This finding is in line with other studies reporting higher scores on the GEFT in individuals with AN compared with controls (Brown et al. 2018; Roberts, Tchanturia, and Treasure 2013). This supports previous speculations of weak central coherence as a candidate for an endophenotype in AN. Given this result, one would have expected a difference in GEFT scores across groups at baseline, but no such difference was detected. A possible explanation for the lower GEFT score at baseline could relate to the acute starvation state where restrictive eating and irregular energy intake might have affected the performance on the test. Alternatively, the improved GEFT scores at follow‐up could reflect practice effects. The RCFT accuracy score was higher in the AN group at follow‐up compared with the baseline results of the COMP group. The performance regarding accuracy may have benefitted from a more global approach. However, regarding the RCFT task, the CCI is considered the most direct measure of central coherence (Lang et al. 2016b). No significant differences in CCI were detected across groups, or within the AN group from baseline to follow‐up. Furthermore, practice effects may have affected the performance in the AN group as the participants had already copied and memorised the Rey figure at the baseline assessment. Unfortunately, as we had no follow‐up data for the COMP group, we could not control for practice effects.

Our results are consistent with some previous studies of adolescents with AN that found no significant difficulties in set‐shifting (Bentz et al. 2017; Shott et al. 2012), and in contrast to other reports of observed set‐shifting impairments in the young AN population (Lang et al. 2015; Wu et al. 2014). The conflicting findings regarding set‐shifting may reflect an association between the duration of illness and cognitive inflexibility. It has been suggested that set‐shifting and central coherence inefficiencies could be a result of enduring illness and long‐term starvation (van Noort et al. 2016). In line with this discussion, Saure et al. (2020) reported no deficits in set‐shifting and central coherence in individuals with short AN duration (defined as < 4 years). Weak central coherence and poor set‐shifting skills were demonstrated in individuals with prolonged AN (defined as > 7 years) (Saure et al. 2020). Our sample consisted of adolescents with a short duration of AN (mean: 14.7 months), which may explain the intact set‐shifting ability compared with the adult population. Due to the greater brain plasticity in younger individuals, it is possible that there is a resilience of neuropsychological traits and related brain systems in the early stages of AN.

Further, we explored the association between illness duration and neurocognitive functioning in our AN group and found that the longer the illness duration, the lower the scores of the object assembly subtest, indicating weaker central coherence. Due to the overall short illness duration in our young AN sample, this association is not likely linked to neurological scarring effects of starvation. The association may suggest weak central coherence being present in a subgroup of adolescents with AN, interfering with recovery and prolonging the illness duration for these individuals.

Examining changes within the AN group, from the acute to the weight‐recovered phase, we found improved performance on the WCST; that is, enhanced set‐shifting ability. However, the performance was not superior to the performance of the comparison group. The improved results are likely explained by practice effects in line with the findings by Basso, Bornstein and Lang; namely, that repeated WCST measurements generate significantly improved scores over a 12‐month period (Basso, Bornstein, and Lang 1999).

Our second hypothesis was partially confirmed as a parent‐child association was found regarding central coherence; on the object assembly test there was a statistically significant correlation between the adolescent females with AN and their fathers. To our knowledge, resemblance in the cognitive profile of adolescents with AN and their fathers has not been reported previously. The father‐child resemblance in central coherence found in the present study supports previous findings of weaker central coherence aggregating in families (Lang, Treasure, and Tchanturia 2016a; Tenconi et al. 2010; Roberts, Tchanturia, and Treasure 2013). In family‐based therapy—the first‐line treatment for adolescents with AN (Monteleone et al. 2022)—parents play a central role in managing and disrupting ED symptoms in their child/adolescent. As the parents are considered an important resource in bringing about recovery in their child, clinicians need to be aware of the cognitive deficits that might be shared by the parent and the child and adapt the interventions accordingly.

In addition, predictors of weight recovery 1 year after baseline assessments were analysed. Results could not confirm that neither weak central coherence nor poor set‐shifting were predictors of a worse outcome (i.e. not weight recovered after 1 year). This result is in line with Keegan et al. (2022) who reported that a higher executive functioning did not predict early change and remission in individuals with AN (Keegan et al. 2022). Age, Z‐BMI and IQ emerged as significant predictors of weight recovery after 1 year. A higher age was a favourable factor. It has been reported previously that an older age at AN onset is a predictor of good long‐term outcome (Dobrescu et al. 2020). A lower Z‐BMI at baseline also predicted weight recovery after 1 year which contrasts other studies showing that a higher BMI at start of treatment is a predictor of good outcome in ED treatment (Vall et al. 2015). The variable importance of both Z‐BMI and age was low according to Tjur's discrimination index. An unexpected finding was that a higher IQ predicted a worse outcome in terms of weight recovery after 1 year, as lower IQ is a known risk factor for mental health disorders. A small number of studies have examined IQ as a predictor of recovery in EDs (Lopez, Stahl, and Tchanturia 2010). Taken together, no firm conclusions could be drawn from these studies, although a descriptive comparison of results implied that individuals recovered from AN had higher scores on IQ measures compared to groups with current AN (Lopez, Stahl, and Tchanturia 2010). One could speculate that individuals with a higher IQ are more driven, goal‐oriented and determined and may use these abilities in favour of the illness, for example by engaging in more persistent behaviours to avoid weight gain. Another possible explanation is that individuals with a higher IQ may be more dedicated to educational achievements and less motivated to prioritise a demanding treatment. However, regarding these possible predictors it has to be taken into account that even though the participants were weight recovered they were not fully recovered from AN. Unfortunately, we did not assess ED symptoms at the 1‐year follow‐up which would have been valuable to evaluate recovery. Future research that is more adequately powered, should explore IQ as a possible predictor of treatment outcome and recovery in AN.

Our third hypothesis could not be confirmed as we found no association between higher levels of ASD/ADHD traits and worse central coherence/set‐shifting performance. An association in the opposite direction was found in the AN‐WR group; the higher the AQ scores, the lower the number of perseverative errors. As discussed above, the greatly improved performance regarding WCST perseverative errors at the reassessment is likely explained by practice effects. We therefore question the reliability of this association. In line with several previous studies (Westwood et al. 2016; Huke et al. 2013; Karjalainen et al. 2019), our results confirmed a higher prevalence of ASD traits in the AN group, as measured with the AQ. Comparable to other reports (Westwood et al. 2016; Karjalainen et al. 2019), our results revealed that the AN group scored significantly higher on the AQ than healthy controls but below the suggested clinical cutoff for ASD. In our AN sample, only one individual scored above the clinical cutoff for ASD after weight recovery. We used a continuous assessment (AQ) to be able to identify individuals with a high AQ score and not only individuals scoring above cutoff. As the female phenotype of ASD might be more difficult to detect, a score just below cutoff is likely clinically relevant in terms of making relevant treatment adaptions.

The prevalence of ASD diagnoses in AN varies widely, and slightly lower rates have been reported in younger populations than in adults (Boltri et al. 2021). As ASD is a pervasive neurodevelopmental disorder with the onset in early childhood, ASD traits need to have been present in early childhood in order to diagnose ASD (American Psychiatric et al.). Based on the DISCO interviews, the presence of childhood traits of ASD could not be confirmed. This might reflect the ASD symptoms arising from the ill state associated with AN and not being present before the onset. Alternatively, parents may not recognise or may underreport symptoms of ASD in their daughters. In recent years, the concept of ‘camouflaging’ has been put forward as one potential explanation. Camouflaging refers to ways in which individuals with ASD mask their symptoms. It comprises behavioural and cognitive coping strategies either to hide behaviours associated with ASD or to engage intentionally in behaviours that are non‐autistic in order to fit in. Camouflaging is often discussed in relation to female gender and could be a partial explanation for the increased rates of missed or late diagnoses found among females (Cook et al. 2021).

To explore traits of ASD further, we examined eating behaviours frequently seen in ASD in the AN group. The more general eating behaviours among the AN group (based on the SWEAA total score) subsided with weight gain. However, the specific eating behaviours frequently seen in ASD (based on the BTSD score) did not decrease significantly after weight recovery, indicating that the adolescents still struggled with social situations at mealtime and simultaneous capacity, such as chewing and cutting the food at the same time. These results are in line with a previous study (Karjalainen et al. 2019) and may indicate that the eating behaviours frequently seen in ASD were already present before the onset of AN. However, regarding both the AQ scores and eating behaviours in our AN group, it has to be taken into account that the majority of the adolescents were not fully recovered from AN at the follow‐up, which may also have influenced the results.

We found a subgroup with a high degree of self‐reported ADHD symptoms (*n* = 4) at the follow‐up, including two individuals who had been assigned a diagnosis. As lower recovery rates have been reported for individuals with a high degree of ADHD symptoms (Svedlund et al. 2018) and as ASD has been linked to longer treatment periods and worse outcome (Saure et al. 2020), it is important to identify these symptoms early in the course of the AN illness. Since parents may not recognise these traits, self‐reports of ASD and ADHD symptoms in adolescents with AN can add valuable information about a patient, highly relevant for clinicians.

### 4.1 Clinical Implications

Identifying the cognitive profile in adolescents with AN has important clinical implications. Interventions can be targeted at improving features of cognitive functioning, such as overfocus on details, for example, by cognitive remediation therapy (CRT) (Tchanturia et al. 2017). Considering the greater brain plasticity in younger individuals, adolescents may be more resilient to the impact of starvation on cognitive function. Interventions targeting these cognitive features could likely have the greatest impact on young individuals. Furthermore, as the cognitive deficits may be shared by family members (Lang, Treasure, and Tchanturia 2016a; Tenconi et al. 2010), playing a central role in the family‐based treatment, we need to gain more knowledge about how the cognitive profile of the parent/s might influence treatment outcome in adolescents. Moreover, the implementation of screening tools into clinical practice to assess traits of ASD and ADHD symptoms in adolescents, who do not respond to treatment, is of high relevance to identify individuals with this comorbidity and guide clinicians. The poorer treatment outcomes in individuals with AN and co‐existent ASD, indicate that they do not respond well to standard treatments (Nimbley et al. 2024) and likely need alternative treatment strategies to recover. One such alternative treatment path is the project Pathway for Eating disorders and Autism derived from Clinical Experience (PEACE) pathway (Tchanturia et al. 2020). The PEACE pathway was developed to provide adapted treatment strategies for individuals with AN and co‐existent ASD and has shown promising results regarding cost‐savings and patients experiences (Tchanturia 2022; Tchanturia et al. 2021).

### 4.2 Strengths and Limitations

The strengths of this study lie in well‐established neurocognitive tests that are mostly used in other studies investigating the cognitive profile in AN, which allow for comparison with published data. As most studies exploring neurocognitive parent‐child resemblance include only mothers, we consider it a strength that we also recruited fathers.

The current study has some obvious limitations. The sample was small which limits the generalisability of the results. Further the sample included only females with AN. The intention was to include males in the AN group but no males fulfiled the inclusion criteria during the time of recruitment. Another limitation is that we did not use a diagnostic interview, such as the MINI KID, to identify psychiatric morbidity in the AN group, but instead relied on registered diagnoses in the medical records of the participants. Furthermore, we did not use a specific eating disorder instrument to assess ED symptoms. A final limitation is that we did not systematically collect data on the subtype of AN (restrictive or binging/purging type), which would have been valuable to explore associations between different subtypes of AN and traits of ASD and ADHD.

## 5 Conclusions

Some support was found for weaker central coherence in adolescents with AN. Together with the father‐child central coherence resemblance, these findings support previous speculations that a typical AN endophenotype includes weak central coherence. Set‐shifting was intact in young females with AN regardless of the phase of the disorder. Traits of autism and ADHD were common in adolescents with AN and not only related to the starvation phase. These traits were not, however, associated with cognitive deficits. Future studies should follow prospectively the cognitive profile and traits of autism and ADHD in larger samples of adolescent onset AN into adulthood.

## Conflicts of Interest

The authors declare no conflicts of interest.

## Supporting information

Table S1

Table S2

Table S3

## Data Availability

The data that support the findings of this study are available from the corresponding author upon reasonable request.
