# Supplementary material for: The Cognitive Profile in Adolescents With Anorexia Nervosa and the Relationship With Autism and ADHD: A Pilot Study
Source: Eur Eat Disord Rev. 2024 Dec 28;33(3):575–88. doi: 10.1002/erv.3168 (PMC11965542; doi:10.1002/erv.3168)
Supplement: Supplementary file 1 — Table S1 [file ERV-33-575-s001.docx]

**TABLE S1. Correlations of neuropsychological measures and self-reports between the adolescents with anorexia nervosa and their parents**

|  | **AN mother**^a^  **(n=15)** | | **AN father**^b^  **(n=16)** | | **AN parents**^c^  **(n=18)** | |
| --- | --- | --- | --- | --- | --- | --- |
| **AN-A group** | r | p | r | p | r | p |
| TMT-4 | -0.275 | 0.320 | 0.178 | 0.508 | -0.147 | 0.558 |
| WCST total score | 0.1842 | 0.546 | -0.187 | 0.521 | 0.083 | 0.751 |
| WCST perseverative responses | -0.037 | 0.902 | -0.1148 | 0.695 | 0.046 | 0.859 |
| WCST perseverative errors | 0.00 | 1.000 | -0.082 | 0.779 | 0.095 | 0.715 |
| WCST number of categories | 0.170 | 0.577 | -0.253 | 0.382 | -0.115 | 0.657 |
| GEFT | 0.055 | 0.843 | 0.115 | 0.671 | 0.062 | 0.806 |
| Object assembly subtest | 0.145 | 0.605 | 0.529 | 0.035 | 0.309 | 0.211 |
| CCI | 0.339 | 0.215 | 0.294 | 0.267 | 0.263 | 0.290 |
|  |  |  |  |  |  |  |
| AQ | 0.1976 | 0.480 | 0.3572 | 0.174 | 0.292 | 0.238 |
| ADHD-RS/ASRS | -0.053 | 0.861 | -0.151 | 0.620 | -0.153 | 0.585 |

AN-A: Acute anorexia nervosa; TMT-4: Trail making test condition 4; WCST: Wisconsin card sorting test; GEFT: Group embedded figures test; CCI: Central coherence index; AQ: the autism spectrum questionnaire. ADHD-RS: The ADHD rating scale IV; ASRS: the ADHD Self Report Scale.

^a^ WCST data were missing for two participants.

^b^ WCST data were missing for two participants.

^c^ Mean value of the scores of the mother and the father.
