# Supplementary material for: The Cognitive Profile in Adolescents With Anorexia Nervosa and the Relationship With Autism and ADHD: A Pilot Study
Source: Eur Eat Disord Rev. 2024 Dec 28;33(3):575–88. doi: 10.1002/erv.3168 (PMC11965542; doi:10.1002/erv.3168)
Supplement: Supplementary file 2 — Table S2 [file ERV-33-575-s002.docx]

**Table S2. Univariable analyses of predictors for weight recovery one year after baseline.**

| **Variable** | **N** | **Odds ratio (95% CI)** | **p** |
| --- | --- | --- | --- |
| Age | 19 | 2.62 (1.02, 6.73) | .045 |
| Z-BMI | 19 | 0.18 (0.03, 1.16) | .07 |
| Duration of AN in months | 13 | 1.08 (0.96, 1.21) | .22 |
| IQ (WASI) | 19 | 0.83 (0.71, 0.98) | .028 |
| TMT-4 | 19 | 1.05 (0.61, 1.82) | .86 |
| WCST perseverative errors | 18 | 1.02 (0.98, 1.06) | .41 |
| GEFT | 19 | 0.90 (0.74, 1.10) | .30 |
| RCFT, central coherence index | 19 | 1.10 (0.11, 10.94) | .94 |
| Object assembly | 19 | 0.79 (0.50, 1.25) | .31 |
| AQ | 19 | 1.01 (0.91, 1.12) | .83 |
| ADHD RS (parent rated) | 16 | 1.33 (0.75, 2.35) | .33 |
| ASSQ (rated by mother) | 12 | 1.09 (0.85, 1.41) | .49 |
| ASSQ (rated by father) | 10 | 1.06 (0.73, 1.53) | .77 |
| SWEAA BTSD score | 17 | 1.01 (0.94, 1.08) | .81 |
| Mother TMT-4 | 15 | 0.93 (0.72, 1.18) | .54 |
| Father TMT-4 | 16 | 1.14 (0.69, 1.89) | .60 |
| Mother WCST perseverative errors | 13 | 1.14 (0.95, 1.37) | .15 |
| Father WCST perseverative errors | 15 | 0.98 (0.88, 1.10) | .75 |
| Mother GEFT | 15 | 0.92 (0.77, 1.10) | .35 |
| Father GEFT | 16 | 0.99 (0.85, 1.16) | .93 |
| Mother RCFT, central coherence index | 15 | 0.33 (0.03, 3.34) | .35 |
| Father RCFT, central coherence index | 16 | 11.45 (0.30, 435.15) | .19 |
| Mother Object assembly | 15 | 0.97 (0.75, 1.26) | .83 |
| Father Object assembly | 16 | 0.96 (0.64, 1.42) | .82 |
| Mother AQ | 15 | 0.95 (0.77, 1.18) | .67 |
| Father AQ | 16 | 0.95 (0.81, 1.13) | .59 |
| Mother ASRS | 15 | 0.99 (0.88, 1.12) | .87 |
| Father ASRS | 16 | 0.97 (0.86, 1.11) | .70 |
| Estimates computed using logistic regression with Firth correction. CI: Confidence interval; BMI: Body mass index; IQ: Intelligence quotient; WASI: Wechsler Abbreviated Scale of Intelligence; TMT-4: Trail making test condition 4; WCST: Wisconsin card sorting test; GEFT: Group embedded figures test; RCFT: Rey Complex figures test; AQ: autism spectrum questionnaire; ADHD-RS: The ADHD rating scale IV; ASSQ: Autism spectrum screening questionnaire; SWEAA BTSD: the Swedish eating assessment for autism spectrum disorders, best two subscale discriminating score. | | | |
