# Supplementary material for: The Cognitive Profile in Adolescents With Anorexia Nervosa and the Relationship With Autism and ADHD: A Pilot Study
Source: Eur Eat Disord Rev. 2024 Dec 28;33(3):575–88. doi: 10.1002/erv.3168 (PMC11965542; doi:10.1002/erv.3168)
Supplement: Supplementary file 3 — Table S3 [file ERV-33-575-s003.docx]

**Table S3. Multivariable analysis of weight recovery one year after baseline including predictors at 0.10 significance in univariable analysis.**

| **Variable** | **Odds ratio (95% CI)** | **p** | **Tjur's  discrimination  index (95% CI)** | **AUC (95% CI)** |
| --- | --- | --- | --- | --- |
| Age | 1.35 (0.34, 5.31) | .67 | 0.14 (-0.01, 0.41) | 0.69 (0.39, 0.99) |
| Z-BMI | 0.19 (0.02, 2.32) | .19 | 0.13 (-0.00, 0.35) | 0.63 (0.34, 0.92) |
| IQ (WASI) | 0.83 (0.69, 1.01) | .058 | 0.32 (0.05, 0.64) | 0.81 (0.53, 1.00) |
| Total |  |  | 0.60 (0.33, 0.81) | 0.82 (0.61, 1.00) |
| Estimates computed using a multivariable logistic model with Firth correction. Tjur's discrimination index is the mean predicted probability of event (i.e., weight recovery) among persons with weight recovery vs non-recovery one year after baseline. AUC is the area under the receiver-operator-curve (ROC) and has been evaluated separately for each univariable predictor and multivariable model using leave-one-out cross validation. CI: Confidence interval; BMI: Body mass index; IQ: Intelligence quotient; WASI: Wechsler Abbreviated Scale of Intelligence. | | | | |
|  | | | | |
| . | | | | |
